# Are current total knee arthroplasty implants tested and approved for personalised alignment?

**DOI:** 10.1002/ksa.12783

**Published:** 2025-07-13

**Authors:** Sabrina Böhle, Leandra Bauer, Matthias Woiczinski, Georg Matziolis

**Affiliations:** ^1^ Orthopaedics, University Hospital Jena, Campus Eisenberg, Waldkliniken Eisenberg Friedrich‐Schiller‐University Jena Jena Germany; ^2^ Experimental Orthopaedics, University Hospital Jena, Campus Eisenberg, Waldkliniken Eisenberg Friedrich‐Schiller‐University Jena Jena Germany

**Keywords:** alignment methods, instruction for use, kinematic alignment, mechanical alignment, TKA

## Abstract

**Purpose:**

Total knee arthroplasty (TKA) traditionally aims for neutral mechanical alignment (MA) to ensure implant longevity. However, this approach ignores individual knee variations, potentially leading to soft‐tissue imbalances and suboptimal functional outcomes. As an alternative, personalised alignment techniques (PAT) have emerged that aim to restore bony anatomy, potentially improve soft tissue balance and thus accelerate recovery. Despite promising short‐term results, long‐term outcomes and regulatory approval remain unclear. This study investigates the extent to which TKA systems from various manufacturers are approved for kinematic alignment and allowable deviations from neutral MA.

**Methods:**

TKA systems were identified using the 2020 Endoprosthesis Registry Germany (EPRD). Manufacturers were systematically contacted to determine if their implants were approved for deviations from neutral MA and the maximum permissible deviations. Follow‐ups in 2021 and 2022 resulted in responses from 11 of 13 manufacturers, with an additional update in 2023 and 2025.

**Results:**

Seven of twelve responding manufacturers either stated that their prostheses were not approved for MA deviations or did not specify details. Among the others, approval varied between implant systems. Two allowed deviations for specific implants without defined limits, while three specified allowable deviation ranges (Triathlon from Stryker with their Mako System 6° varus to 3° valgus; ATTUNE CR FB from Johnson & Johnson deviation 7° varus to 3° valgus°; ATTUNE PS FB and ATTUNE RP ± 3°; Primary system from Zimmer Biomet 0 ± 5° with their ROSA system). Revision systems universally restricted axis deviation due to mechanical constraints.

**Conclusion:**

Only a limited number of manufacturers have conducted rigorous testing of their systems for deviations from MA, and consequently, are able to specify limits to such deviations. Additionally, practical limitations arise from the complexity of PAT, patient‐specific factors and prosthesis suitability. To safeguard patient safety and ensure implant longevity, manufacturers should systematically evaluate alignment deviations through standardised tests or computational simulations.

**Level of Evidence:**

N/A.

**Abbreviations**
AA: anatomic alignment
aMA: adjusted mechanical alignment
EPRD: Endoprosthesis Registry Germany
FA: functional alignment
iKA: inverse kinematic alignment
KA: kinematic alignment
MA: mechanical alignment
PAT: personlized alignment techniques
rKA: restricted kinematic alignment
TKA: total knee arthroplasty

## INTRODUCTION

For decades, the primary goal in total knee arthroplasty (TKA) has been to achieve a neutral mechanical axis (MA), defined as a hip–knee–ankle (HKA) angle of 0°, with a permissible range of ±3° [38]. This alignment strategy was initially adopted to optimise implant survival rates. However, this does not take individual variations in knee joint axes into account. Consequently, this approach may lead to soft‐tissue imbalances, particularly when the joint line is not considered. Despite following this alignment strategy, a significant number of patients (10%–20%) continue to report dissatisfaction with their functional outcomes [4, 6, 23]. This raises the question of how individual patient characteristics can be more effectively incorporated into the alignment process. To address these limitations, several personalised alignment techniques (PAT) have been developed, including kinematic alignment (KA), restricted kinematic alignment (rKA), inverse kinematic alignment (iKA) and functional alignment (FA) [15].

KA aims to restore the prearthritic state by aligning prosthetic components according to the patient's individual bony anatomy, thereby often reducing the need for extensive soft‐tissue releases compared to traditional MA [1, 2, 27]. Following on from the classic KA without limiting the deviation from the MA, the rKA was developed. This technique aims to limit extreme implant positioning within a range, typically around ±3° of the HKA angle, by first adjusting the femoral component followed subsequently by the tibial component. iKA, on the other hand, prioritises the restoration of the tibial joint line, with the femoral component adjusted thereafter [15, 34]. FA emphasises implant positioning that minimally compromises the surrounding soft‐tissue envelope, thus restoring the joint's plane and obliquity according to soft‐tissue constraints [29]. Furthermore, the ‘tibia‐first, gap‐balanced’ patient‐specific alignment technique involves initially performing a tibial resection and subsequently balancing the knee joint gaps to individually restore bony morphology and joint line obliquity in varus, neutral and valgus knees [11].

Research by Rivière et al. comparing MA with PAT underscored the advantages of KA concerning early functional outcomes [5, 7] and faster recovery [8]. Nonetheless, the authors highlighted the importance of cautious patient selection and extended longitudinal analysis to confirm sustained benefits [33]. Although further studies confirmed a trend towards improved outcomes with PAT, they advocated a cautious transition from MA [15]. In addition, significant benefits were shown when using KA for certain knee phenotypes, particularly emphasising better soft tissue balance for certain types of alignment in the coronal plane [17]. Moreover, Graichen et al. introduced the knee SIPR scoring system, demonstrating quantitatively through a computational TKA simulator that PAT, particularly using measured‐resection and gap‐balancing techniques, outperformed traditional MA in restoring knee anatomy and joint laxity, especially in varus and valgus phenotypes [12]. Ettinger et al. further provided clinical evidence favouring rKA over MA, specifically regarding patient satisfaction, joint awareness, and functional outcomes in patients with varus knee types [9]. Shatrov et al. found that KA failed to achieve balanced knees in over 50% of varus cases, particularly in the flexion gap, and resulted in significantly greater bone resection compared to FA [36]. Some other studies have indicated that PAT TKA may result in superior functional outcomes [24, 25, 26, 35, 40]. However, varus angles exceeding 6° have been associated with diminished clinical outcomes [28, 40].

Positioning of the implant according to various PAT must be clearly distinguished from malalignment, which is defined by undesired incorrect positioning. A study that explicitly examined the survival rate of KA—as a form of PAT—TKA demonstrated that the implant survival rate was 98.4% at the 10‐year follow‐up [14]. However, a review of the literature on TKA malalignment revealed that revision rates are higher for TKA malalignment than for neutral alignment [10, 32]. Additionally, malrotation of one component, for example, tibial varus alignment has been identified as a risk factor for higher revision rates compared to neutral or valgus tibial components [16, 30, 32]. Conversely, several studies have not identified a significant difference in revision rates between malaligned and well‐aligned TKAs [3, 18, 19, 31].

Given the ongoing debates and variability in results concerning functional outcomes and implant longevity, the approval process for TKA systems becomes a critical consideration. This raises the question of whether implant manufacturers explicitly test or permit deviations from the neutral MA during their approval processes. Thus, the current study aims to evaluate various TKA systems from multiple manufacturers regarding their official approval for PAT applications and determine permissible MA deviations. The aim of this study is to show for the first time which TKA systems from various manufacturers are approved for a PAT and whether a maximum deviation from a neutral MA is permitted.

## MATERIALS AND METHODS

This study was based on data from the annual report 2020 of the Endoprosthesis Registry Germany (EPRD), which was used to identify all TKA systems implanted in Germany.

Manufacturers of these systems were contacted via post or email to inquire about the permissibility of alternative alignment techniques for their implants. The inquiry comprised two specific questions:

1. Are the following implants from your company approved for implantation with deviations from a mechanically straight leg axis (varus/valgus) in accordance with regulatory approval?
2. If yes, what is the maximum allowable deviation (varus/valgus)?

Each manufacturer was provided with a table listing the identified TKA systems associated with their company. The table included columns for the responses to ‘Deviation allowed?’ and ‘Maximum allowable deviation’. This structured format facilitated the systematic collection of information regarding alignment flexibility and regulatory constraints for each system.

The companies were initially contacted by post in mid‐May 2021, and a total of seven companies responded. In December 2021, a new inquiry was sent by email to the companies that had not yet responded. A final inquiry was sent again in July/August 2022 by email or contact form from the respective companies, resulting in eleven responses from a total of 13 companies.

An update was carried out with the EPRD 2023, whereby one company with a new system and three additional companies were contacted.

In addition, in March 2025, all manufacturers received a summary of their data for correction and checking for up‐to‐dateness and correctness, which means that the data given in the following results section can be treated as of 03/2025.

## RESULTS

Out of the 13 manufacturers contacted between 05/2021 and 08/2022, 11 manufacturers responded to the inquiry. Additionally, one out of three additional companies has been added in 12/2024 and 01/2025. No feedback or information were received from three companies. All manufacturers listed in Table 1 responded to the information in the results table and adjusted their initial information where necessary (03/2025).

**Table 1:** Overview of total knee arthroplasty company approval for allowed deviation from mechanical alignment.

Implant manufacturer	Modell	Deviation allowed?	Maximal deviation
Aesculap AG (B. Braun Deutschland GmbH & Co. KG)	VEGA	Not defined	n. a.
	e.motion	Not defined	n. a.
	Columbus	Not defined	n. a.
	Columbus Revision	Not defined	n. a.
	EnduRo	Not defined	n. a.
Implantcast GmbH	ACS FB	Not defined	n. a.
	ACS MB	Not defined	n. a.
	ACS FB SC	Not defined	n. a.
	ACS MB SC	Not defined	n. a.
	5C® Knee System	Not defined	n. a.
	MUTARS	Not defined	n. a.
Johnson & Johnson Medical GmbH	ATTUNE CR FB cemented/cementless (CR and MS)	Yes	HKA −7° (varus) to 3° (valgus) Tibia −7° (varus) to 0°
	ATTUNE PS FB cemented/cementless	Yes	HKA −3° (varus) to 3° (valgus) Tibia −3° (varus) to 0°
	ATTUNE RP cemented/cementless	Yes	HKA −3° (varus) to 3° (valgus) Tibia −3° (varus) to 0°
	LCS COMPLETE	No	0°
	PFC Sigma	No	0°
	Sigma TC3	No	0°
	ATTUNE Revision	No	0°
	LCS complete Revision	No	0°
	S‐Rom Noiles	No	0°
Mathys AG	balanSys BICONDYLAR	Yes	Not defined
	balanSys REVISION	No	0°
Medacta Germany GmbH	GMK Sphere/SpheriKA	Yes, following the validated and approved kinematic alignment (KA) technique	No restrictions if the validated KA technique is applied
	GMK Primary	No	n. a.
	GMK Revision	No	n. a.
	GMK Hinge	No	n. a.
OHST Medizintechnik AG	ZEN	No	n. a.
	EFK	No	n. a.
Peter Brehm GmbH	BPK‐S Integration (primary)	Not defined	n. a.
	BPK‐S Integration (revision)	No	n. a.
Smith & Nephew GmbH	JOUNREY II TKA	Not defined	n. a.
	LEGION	Not defined	n. a.
	GENESIS II	Not defined	n. a.
	TC‐Plus Primary	Not defined	n. a.
	TC Primary S‐Line	Not defined	n. a.
	LEGION Revision	Not defined	n. a.
	RT Plus R Plus Modular	Not defined	n. a.
Speetec GmbH	Empowr 3D Knee	No	n. a.
	3D Knee (Classic)	No	n. a.
	Exprt Precision System‐Revision Knee	No	n. a.
Stryker GmbH & Co. KG	Triathlon	Yes, only with Mako system	n. a.; with Mako preoperative plan 3° valgus—6° varus °
	Triathlon TS	Not defined	n. a.
Waldemar Link GmbH & Co. KG	GEMINI SL	Not defined	n. a.
	LCK Link Classis	Not defined	n. a.
	Endo‐Model	Not defined	n. a.
	LinkSymphoKnee	Not defined	n. a.
Zimmer Biomet Germany GmbH	Persona	Yes, only with ROSA system	0 ± 5°
	Persona System USA, Surgical technique Kinematic Alignment	Yes	0 ± 5°
	Vanguard Knee System	Yes, only with ROSA system	0 ± 5°
	NexGen	Yes, only with ROSA system	0 ± 5°
	Innex	No	n. a.
	Natural Knee	No	n. a.
	NexGen Constrained Condylar Knee (LCCK)	No	n. a.
	NexGen Rotating Hinge Knee	No	n. a.
	Vanguard 360° Revision Knee System	No	n. a.

### Manufacturer with defined information on the deviation from the MA in the corona plane

Johnson & Johnson Medical GmbH, the manufacturer of the ATTUNE knee prosthesis, provides specific guidelines regarding alignment tolerances for its various implant systems. For the cemented/cementless ATTUNE CR FB prosthesis with a CR or MS inlay, a deviation of −7° to 3° HKA is permitted, whereby the tibia may deviate 0°–7° varus. For the cemented/cementless ATTUNE PS FB and RP, a deviation of ±3° is approved, with a tibial deviation of 0°–3° varus.

Mathys AG provided the following information regarding their systems: For the BalanSys BICONDYLAR system, the recommended surgical technique is based on soft tissue tension, achieved using a calibrated dual spring tensioner. This approach does not explicitly reference alignment to the frontal plane, suggesting a soft tissue‐based alignment method. Additionally, a ‘Combination Technique’ is described, which combines axis‐guided alignment with soft tissue balancing [13].

Stryker GmbH & Co. KG reported that their primary system is cleared for a Mako preoperative plan of 3° valgus to 6° varus. They also state, ‘A surgeon may choose to place components outside the preoperative planning guidelines based on the clinical needs of the patient.’

Zimmer Biomet Germany GmbH mandates MA as the standard for all its implant systems. The primary objective is to achieve adequate soft tissue balancing and optimal joint stability. An exception exists for the Persona system in the United States, where surgical guidelines include an option for KA, referencing Howell's technique. Finally, the information provided by Zimmer Biomet Germany GmbH emphasises its surgical technique [43]. These tools leave the determination of specific surgical goals and alignment methods to the discretion of the operating surgeon. The situation is different for operations with the ROSA system, which is approved for use with the Persona Knee System, Vanguard Knee System and NexGen Knee System. Here, a 5° ± varus‐valgus adaptation and a 3° internal to 10° external rotation of the femoral component is permitted. Approval of the Persona Knee System for a nonrobotic application is planned.

### Manufacturer without defined information on the deviation from the MA in the corona plane

All the systems from Aesculap AG, Implantcast GmbH, OHST Medizintechnik AG, Peter Brehm GmbH, Smith & Nephew GmbH, Speetec GmbH and Waldemar Link GmbH & Co. KG, no deviations from the MA have been defined (not defined) or are not permitted (no). All Johnson & Johnson systems except the ATTUNE FB cemented, from Mathys AG the revision system, from Stryker GmbH & Co. KG the Triathlon TS, and from Zimmer the Innex, Natural Knee, NexGen Constrained Condylar Knee (LCCK), NexGen Rotating Hinge Knee as well as Vanguard 360° Revision Knee System are not permitted for a deviation (see Table 1).

For a more detailed description for undefined or missing permission to deviate from the MA, please refer to the File S1.

## DISCUSSION

The most important finding of the present study was 7 of 12 manufactures either stated that their prostheses were not approved for MA deviations or did not specify details. Of the other five companies, two allowed deviations for specific implants without defined limits, while three companies specified allowable deviation ranges (Triathlon from Stryker with their Mako System 6° varus to 3° valgus; ATTUNE CR FB from Johnson & Johnson deviation 7° varus to 3° valgus°; ATTUNE PS FB and ATTUNE RP ± 3°; Primary system from Zimmer Biomet 0 ± 5° with their ROSA system).

The specific mechanical testing conditions of the knee implants used for CE certification are not fully disclosed, as parts of the design validation may involve manufacturer‐specific, nonstandardised protocols. In general, mechanical evaluations follow international standards such as ISO 14243 (wear testing), which prescribe load profiles based on a neutral MA but do not account for patient‐specific variables such as increased knee adduction moments (KAMs) or deviations in dynamic limb alignment.

A notable finding of the survey among implant manufacturers is the frequent lack of precise information regarding the suitability of their knee prostheses for PAT. Many manufacturers emphasise that the decision ultimately rests with the operating surgeon. This approach may suggest a strategic effort to safeguard themselves from legal repercussions. However, it raises critical questions about the practical implications for surgeons: how should they proceed if patient‐specific anatomy favours a PAT, but the implants in use are strictly approved or tested for alignment based on the MA or the manufacturer makes no statement regarding PAT? The aforementioned uncertainties present a challenging predicament for surgeons, who must navigate the complexities of patient‐specific needs while adhering to the constraints of regulatory and legal frameworks.

The predominance of MA in implant system testing reflects its status as the gold standard for regulatory approval. Transitioning from this conventional standard to accommodate PAT would require comprehensive new testing protocols, including biomechanical load evaluations under PAT conditions. Such testing is both time‐intensive and costly, potentially discouraging manufacturers from pursuing formal approval for PAT. In addition, the lack of standardised regulatory guidelines for PAT exacerbates the uncertainty surrounding its adoption. Without clear directives from regulatory bodies, manufacturers may be reluctant to invest in the necessary research and development required to validate their implants for PAT.

Deviation from MA raises additional concerns about unexpected stresses on the prosthesis, which could increase the risk of premature failure. Changes in frontal plane alignment influence the KAM in dynamic conditions, such as walking. KAM arises from the ground reaction force acting in the frontal plane during gait. Several studies have examined the relationship between KAM and the distribution of forces on the medial and lateral condyles in total TKA. Trepczynski et al. conducted a study, including activities like walking, standing up and descending stairs. Averaging across patients and activities, they observed a strong correlation (*R*² = 0.88) between the KAM and medial contact force [39]. In particular, varus alignment leads to a higher KAM due to the lengthened lever arms. Srivastava et al. were able to show in vivo on explants that varus implanted tibial components, which have an increased KAM, exhibit significantly higher wear [37]. A study by Moyer et al. demonstrated that a one‐degree varus alignment increment resulted in a 3.2 Nm augmentation in the KAM, thereby exerting a substantial influence on the load distribution. This may potentially lead to overloading of the polyethylene components over an extended period [22]. These findings raise concerns about whether current implant materials and designs are sufficiently robust to withstand altered loading conditions induced by PAT without increasing the risk of failure. Further investigations into material properties, wear resistance and long‐term biomechanical stability under PAT conditions are therefore necessary.

In silico and finite element studies have shown that implant alignment has a significant impact on biomechanical loading and long‐term wear. Mell et al. analysed nine different alignment parameters and demonstrated that optimised component placement in the transverse plane can significantly reduce wear [21]. These findings highlight the need to explicitly investigate the wear resistance of implants with PAT, as deviations from the MA lead to altered loading patterns that may increase polyethylene wear and the risk of implant failure. Zheng et al. demonstrated that even minimal translational deviations of the tibial platform can lead to significant biomechanical changes [42]. These findings highlight the importance of precise planning and placement techniques, particularly in the context of the PAT.

A key factor contributing to these loading differences is individual anatomical variation. Willems et al. demonstrated that differences in tibial and femoral morphology significantly affect load distribution in the medial compartment [41]. This is particularly relevant for PAT, as patient‐specific implant placement may potentially achieve a more physiological load distribution compared to standardised MA alignment. However, this requires more detailed biomechanical evaluations to ensure that PAT does not lead to unintended overloads in specific patient groups.

Finite element models provide a valuable methodology for further analysing such loading patterns. Mebarki et al. developed a model to evaluate patellofemoral forces and stresses during squatting after posterior‐stabilised TKA [20]. Applying this methodology to PAT could help identify specific load peaks and better understand their potential impact on implant long‐term stability. This underscores the necessity of developing PAT specific in silico models to gain well‐founded insights into the biomechanical effects of this alignment strategy.

Manufacturers may fear that these complications could elevate their liability exposure and render their products more vulnerable to legal claims. As a result, it is likely that manufacturers would require explicit clinical evidence demonstrating noninferiority—or potential superiority—of PAT compared to MA before pursuing regulatory approval for PAT‐specific implants. However, the lack of long‐term, large‐scale outcome studies remains a critical barrier.

These considerations underscore the necessity for more rigorous biomechanical studies that assess prosthesis performance under PAT conditions. In particular, a standardised test battery incorporating in vivo, in vitro and in silico analyses could provide a more comprehensive evaluation of implant performance under PAT conditions. Such standardised testing frameworks could also aid in identifying patient populations for whom PAT may be particularly beneficial, thereby enabling a more targeted approach in clinical decision‐making. It therefore makes sense to develop new test standards for implant approval that ensure a more evidence‐based approach to assessing the feasibility of PAT. Furthermore, the establishment of uniform testing protocols for PAT could alleviate manufacturer concerns and facilitate broader adoption.

Several limitations must be considered when interpreting the results of this study. First, despite multiple attempts at contact, two manufacturers did not respond, potentially introducing response bias and limiting the comprehensiveness of our findings. Additionally, the reliance on manufacturers' self‐reported regulatory information without independent verification may impact the accuracy and objectivity of the collected data. Furthermore, the structured survey method, while effective for systematic data gathering, may not capture nuanced explanations or rationales behind alignment deviations, thus limiting qualitative insights into manufacturers' practices or regulatory reasoning. The timing and frequency of follow‐up inquiries might also have influenced response rates and data completeness, as companies may have updated their approval criteria between inquiries. Lastly, since the study was specifically based on data from the German Endoprosthesis Registry, findings may have limited generalisability outside the German healthcare context, as regulatory standards and practices vary internationally.

While early studies have demonstrated functional advantages of PAT, as KA and rKA, the long‐term outcomes, particularly with respect to implant survival rates, remain inconclusive. This uncertainty further complicates the integration of PAT into routine clinical practice and regulatory frameworks, highlighting a need for more extensive research to establish its viability as an alternative to MA. Future clinical trials should therefore focus not only on functional outcomes but also on implant longevity, patient satisfaction and complication rates to determine whether PAT can be a sustainable alternative in the long term. The integration of registry data could provide additional insights into real‐world performance, supporting evidence‐based decision‐making.

## CONCLUSION

In conclusion, it can be stated that only a limited number of manufacturers have conducted rigorous testing of their systems for deviations from MA, and consequently, are able to specify limits to such deviations. At the same time, the practical limitations of PAT arise from the complexity of the approach, patient‐specific factors, and the suitability of prosthesis designs. To fully realise the potential of PAT, standardised testing protocols, a stronger evidence base and advancements in implant technology are necessary. Only through these measures can it be ensured that this innovative approach provides functional benefits for patients without compromising the safety and durability of the implants.

## AUTHOR CONTRIBUTIONS

**Sabrina Böhle**: Conceptualisation; methodology; data analysis; formal analysis and investigation; writing—original draft preparation. **Leandra Bauer**: data analysis; formal analysis and investigation; writing—original draft preparation. **Matthias Woiczinski**: Writing—review and editing; supervision. **Georg Matziolis**: conceptualisation; methodology; writing—review and editing; supervision.

## CONFLICT OF INTEREST STATEMENT

The authors declare no conflicts of interest.

## ETHICS STATEMENT

No experiments were carried out on humans or animals, so no ethics vote was required.

## Supporting information

SupInformation.

## Data Availability

The data that support the findings of this study are available from the corresponding author upon reasonable request.
