# Supplementary material for: Are current total knee arthroplasty implants tested and approved for personalised alignment?
Source: Knee Surg Sports Traumatol Arthrosc. 2025 Jul 13;34(1):174–82. doi: 10.1002/ksa.12783 (PMC12747626; doi:10.1002/ksa.12783)
Supplement: Supplementary file 1 — SupInformation. [file KSA-34-174-s001.docx]

**Supplementary Information**

- 1. Aesculap AG (B. Braun Deutschland GmbH & Co. KG)

The company has not defined any deviation from the standard MA alignment and consequently no maximum deviation. The regulatory approval process does not define the alignment of the components. The components have been tested in an MA configuration for 40 years.

- 1. Implantcast GmbH

The manufacturer Implantcast GmbH indicated that the IFU for the ACS® Knee System do not specify the alignment, making it impossible to define maximum deviations. All tests were conducted in a neutral alignment, and no additional data are available. However, they do not recommend the implantation of cementless tibial components in posterior-stabilized (PS) designs for cases with moderate to severe varus deformities. For the stem-guided, semi-constrained and rotating hinge revision systems (ACS MB SC, ACS FB SC, and MUTARS Knee System), a deviation from the mechanical axis is not recommended. For the 5C^®^ Knee System, no statement can currently be made regarding possible axis deviation. However, initial tests for the approval of defined axis deviations have been conducted with positive results. Further testing is planned to obtain approval, at least for restricted KA.

- 1. Johnson & Johnson Medical GmbH

Johnson & Johnson Medical GmbH, the manufacturer of the ATTUNE knee prosthesis, provides specific guidelines regarding alignment tolerances for its various implant systems. For the cemented/cementless ATTUNE CR FB prosthesis with a CR or MS inlay, a deviation of -7° to 3° HKA is permitted, whereby the tibia may deviate 0-7° varus. For the cemented/cementless ATTUNE PS FB and RP, a deviation of ±3° is approved, with a tibial deviation of 0-3° varus. All other Johnson & Johnson systems, require strict adherence to the mechanical axis with no allowable deviations.

- 1. Mathys AG (now a company of enovis)

Mathys AG provided the following information regarding their systems: For the BalanSys BICONDYLAR system, the recommended surgical technique is based on soft tissue tension, achieved using a calibrated dual spring tensioner. This approach does not explicitly reference alignment to the frontal plane, suggesting a soft tissue-based alignment method. Additionally, a "Combination Technique" is described, which combines axis-guided alignment with soft tissue balancing [12].

For their revision systems, only axis-guided alignment is feasible due to the integration of implants with stems. The alignment is determined by the intramedullary anatomy in these cases.

- 1. Medacta Germany GmbH

Medacta Germany GmbH reported that for system GMK Sphere/SpheriKA, deviations from the mechanical axis are permitted without restrictions if the validated and approved KA technique is followed. The other systems are not permitted to deviate from the mechanical axis in the IFU: Even though a tolerance of ±3° normally applies, the tests were limited to 0° in an MA scenario.

- 1. OHST Medizintechnik AG

OHST Medizintechnik AG stated that, the aim for both the ZEN and EFK knee systems is to achieve a neutral mechanical leg axis (0°). Deviations are not recommended. There is no evidence up to which deviation from this axis allows safe use of these knee systems.

- 1. Peter Brehm GmbH

Peter Brehm GmbH regulatory approved the BPK-S Integration Knee System based on state of the art testing methods in neutral alignment. There is no data available regarding implant performance under deviations from this alignment setting. However, deviations from this neutral alignment are not regulatory approved or recommended within the instruction for use.

- 1. Smith & Nephew GmbH

Smith & Nephew GmbH reported that their systems are regulatory approved exclusively for mechanical alignment. Deviations from this alignment method are not certified and are currently not yet released.

- 1. Speetec GmbH (now Mathys AG, a company of enovis)

Speetec GmbH stated, that all primary 3D knee implants are tested for material durability and wear resistance based on a neutral mechanical axis. A deviation from the mechanically straight leg axis in the femur results only from the instrumentally specified permissible valgus angles; femorally, the 3D (Classic) model is set to a valgus angle alignment between 5°, 6° and 7° in relation to the anatomical axis determined intramedullary. With the EMPOWR 3D model, this selection option is 2° to 8° valgus in 1° increments.

The mechanical axis also determines the frontal tibial alignment.

With the ‘Exprt Precision System-Revision Knee’, the implant stems define the alignment of the implants on the femoral and tibial side, as well as according to the surgical technique, in relation to the axes of the respective intramedullary situation. On the femoral side, the modular stems are fixed to the implant at 5° valgus in relation to the joint line. Tibially, the stems are fixed perpendicular to the joint line. Provided that the stems are implanted in the correct axis, deviation is therefore not possible.

- 1. Stryker GmbH & Co. KG

Stryker GmbH & Co. KG reported that their primary system is cleared for a Mako pre-operative plan of 3° valgus to 6° varus. They also state, “A surgeon may choose to place components outside the pre-operative planning guidelines based on the clinical needs of the patient.”  However, their revision system does not allow deviation from the mechanical axis as this is dictated by the stems.

- 1. Waldemar Link GmbH & Co. KG

Waldemar Link GmbH & Co. KG clarified that the approval process for their systems was based on testing conducted in neutral alignment, and no data are available regarding performance under deviations from this alignment.

For unconstrained systems, no technical statements can be made regarding alignment deviations. In contrast, deviations from the mechanical axis are explicitly not recommended in constrained systems resulting from their constrain mechanism. In case of a deviation from the MA, the manufacturer recommends opting for the next level of implant that offers increased stability.

- 1. Zimmer Biomet Germany GmbH

Zimmer Biomet Germany GmbH mandates mechanical alignment as the standard for all its implant systems. The primary objective is to achieve adequate soft tissue balancing and optimal joint stability. An exception exists for the Persona system in the United States, where surgical guidelines include an option for kinematic alignment, referencing Howell's technique. However, this approach does not apply to other implant systems from the manufacturer. For revision systems, Zimmer Biomet Germany GmbH employs linked components, which are technically constrained to a 0° alignment in the frontal plane, precluding any alternative alignment methods.

Finally, the information provided by Zimmer Biomet Germany GmbH emphasizes its surgical technique [40]. These tools leave the determination of specific surgical goals and alignment methods to the discretion of the operating surgeon.

The situation is different for operations with the ROSA system, which is approved for use with the Persona Knee System, Vanguard Knee System and NexGen Knee System. Here, a 5° ± varus-valgus adaptation and a 3° internal to 10° external rotation of the femoral component is permitted. Approval of the Persona Knee System for a non-robotic application is planned.
